# Catalytic DNA for Precision Diagnostics: DNAzyme‐Based Approaches to Single‐Nucleotide Polymorphism Detection

**DOI:** 10.1002/cbic.70591

**Published:** 2026-11-05

**Authors:** Marah Alkhalili, Saqer Al Abdullah, Farbod Ebrahimi, Kristen Dellinger

**Affiliations:** ^1^ Department of Nanoengineering Joint School of Nanoscience and Nanoengineering North Carolina A&T State University Greensboro North Carolina USA; ^2^ Department of Biochemistry, Translational Eye and Vision Research Center (TrEVR) Wake Forest School of Medicine Winston‐Salem North Carolina USA

**Keywords:** DNAzymes, molecular diagnostics, nucleic acid biosensors, signal amplification, SNP detection

## Abstract

Single‐nucleotide polymorphisms (SNPs) are the most abundant form of human genetic variation and serve as important biomarkers in disease risk assessment, therapeutic stratification, and precision medicine. Yet accurate detection of low‐abundance SNPs in complex biological samples remains difficult with many conventional genotyping methods, which often rely on sophisticated instrumentation and show limited tolerance to matrix interference. DNAzyme‐based biosensors offer a promising alternative by coupling programmable nucleic acid recognition with catalytic signal generation under isothermal conditions. This review highlights recent advances in DNAzyme‐based SNP detection across optical, electrochemical, and amplification‐assisted formats, including fluorescence, colorimetric, chemiluminescent, rolling circle amplification, loop‐mediated isothermal amplification, hybridization chain reaction, and catalytic hairpin assembly strategies. We also discuss molecular design principles that enhance single‐base discrimination, such as binding‐arm engineering, cofactor tuning, and computational sequence design. Finally, we outline current limitations, including nuclease susceptibility, temperature dependence, and performance in complex sample matrices, and consider emerging opportunities for DNAzyme‐enabled SNP analysis in point‐of‐care diagnostics, personalized medicine, and large‐scale genomics.

## Graphical abstract

This review covers DNAzyme sensor architectures for single‐nucleotide polymorphism (SNP) genotyping, including gold nanoparticle (AuNP) and amplification‐assisted formats, and the design rules governing single‐base discrimination.

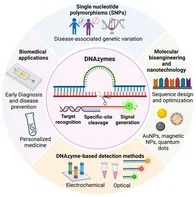

## 1 Introduction

Variation at a single‐nucleotide position is the most common form of genetic difference between individuals, and such polymorphisms are central to disease association studies, pharmacogenomics, and precision medicine [1, 2]. From a detection perspective, however, single‐nucleotide polymorphism (SNP) genotyping remains challenging because it requires reliable discrimination between nucleic acid sequences that differ by only a single base. In practical assay settings, this small sequence difference can be difficult to convert into a large and reproducible signal contrast, especially in formats intended for rapid or low‐complexity analysis [3, 4]. Conventional SNP genotyping methods, including polymerase chain reaction (PCR)‐based assays and restriction fragment length polymorphism (RFLP) analysis, are well‐established analytical techniques for variant detection [2, 3, 5]. However, many established workflows rely on enzymes, thermal cycling, multistep handling, and instrument‐intensive readouts, and this has motivated the development of isothermal and chemically programmable sensing strategies for nucleic acid analysis [6, 8]. Such approaches are particularly attractive in biosensing applications where molecular recognition can be translated into a simple optical or electrochemical output.

Nowadays, considerable effort has been devoted to designing new sensors with high specificity for SNP detection. For example, OWL sensors are multicomponent DNA nanostructures that require a fully complementary target DNA or RNA to generate a detectable signal [9]. In these sensors, a single mismatch can cause destabilization in the assembled OWL structure in a way that significantly suppresses the signal generation [9]. Furthermore, multicomponent hybridization probes have been engineered in which two or more probe molecules bind to different regions of the target and form junctions or higher‐order complexes [10]. In such systems, the targets act as a scaffold that brings all the components together and generates the signal, whereas a single polymorphism in the target causes a mismatch and indeed disrupts the complex formation and signal production [10]. In fact, these noncatalytic hybridization approaches have the potential to provide highly specific designs for SNP detection. However, their multicomponent structures increase the assay and design complexity, which requires optimization of multiple components, probe assembly, and hybridization conditions. In fact, these efforts highlight the continued need to develop new sensors for the detection of the SNP and suggest that different sensing strategies may serve as complementary approaches rather than alternatives to one another.

DNAzymes are especially promising scaffolds due to their ability to combine the sequence programmability of nucleic acids with catalytic signal transduction [11, 12]. In fact, a single activated DNAzyme has the potential to catalyze the cleavage of multiple substrate molecules [13]. In other words, this enables multiple catalytic turnovers under suitable conditions. This repeated catalytic activity allows the DNAzyme to amplify the endpoint signal, such as optical or electrochemical, without the need for target amplification [13, 14]. The signal amplification ability of DNAzyme makes it an interesting candidate to be integrated with different signal‐generation platforms. For example, DNAzyme can be coupled with different organic fluorophore–quencher systems or quantum dots. Another example is functionalizing the DNAzymes to gold nanoparticles (AuNPs) in colorimetric sensors, which show blue color when the particles aggregate and red when they are stabilized by substrate cleavage.

A highly important advantage of the utilization of DNAzymes in nucleic acid biosensing is their ability to provide signal amplification in addition to target amplification [15, 16]. The nucleic acid amplification methods, like PCR, have the potential to increase the number of copies of the target sequence; on the other hand, DNAzyme‐mediated amplification enhances the signal generated from the amplified target via catalytic substrate conversion. This can be highly important, especially when the amount of amplified product is still too low to generate a sufficient signal‐to‐noise ratio. Unlike noncatalytic probes, such as molecular beacons, in which target hybridization produces a stoichiometric fluorescence response, DNAzymes can catalyze substrate cleavage and, in suitable assay designs, undergo multiple catalytic turnovers. In fact, this catalytic feature can improve analytical sensitivity and the signal‐to‐background ratio, particularly when detecting low‐abundance amplicons.

The ability of DNAzyme to amplify the signal without the need for target amplification provides simple sensor platforms with reduced instrumentation and workflow complexity [17]. However, in the absence of target amplification, the sensitivity of DNAzyme‐based assays depends strongly on DNAzyme architecture, catalytic efficiency, target accessibility, and reaction conditions. In contrast, the analytical sensitivity of techniques like PCR or qPCR relies on the amplification of the target sequence, although their performance can be affected by nonspecific amplification, primer‐dimer formation, and suboptimal reaction conditions. Also, they require expensive instrumentation. Further, DNAzymes can also achieve the high specificity of differentiating a SNP by optimizing the recognition‐arm design and the mismatch positioning. Similarly, methods like CRISPR/Cas provide highly programmable sequence recognition and can achieve single‐nucleotide discrimination, but many CRISPR formats still heavily rely on preamplification or additional signal enhancement, which can increase assay complexity and reagent requirements [18]. Therefore, DNAzyme sensing methods may be advantageous when simplified instrumentation and catalytic signal generation are prioritized.

RNA‐cleaving DNAzymes can couple target recognition to substrate cleavage and signal amplification, whereas peroxidase‐mimicking G‐quadruplex DNAzymes provide an alternative route to colorimetric, chemiluminescent, and electrochemical readout [19, 20, 21]. Their low synthetic cost, thermal stability, and compatibility with modular nucleic acid circuitry have made DNAzymes versatile components in biosensor design [11, 12].

Yet the analytical performance of DNAzyme‐based SNP assays depends not only on the catalytic motif itself but also on probe architecture, binding‐arm design, mismatch position, target accessibility, secondary structure, cofactor conditions, and the integration of gating or amplification strategies [4, 11, 20]. Recent studies have further shown that coupling DNAzyme transduction with isothermal amplification modules such as catalytic hairpin assembly (CHA), hybridization chain reaction (HCR), rolling circle amplification (RCA), and related circuits can improve sensitivity while preserving programmable recognition [20, 22]. More selective single‐base discrimination can also be achieved through competitive or protected hybridization designs that penalize mismatched targets during strand displacement and branch migration [4, 23]. This review therefore examines how DNAzyme chemistry, signal transduction mode, and molecular engineering strategy together govern sensitivity and selectivity in SNP detection, with emphasis on optical, electrochemical, and amplification‐assisted platforms relevant to chemical biology and biosensing (see Figure 1).

**FIGURE 1:**
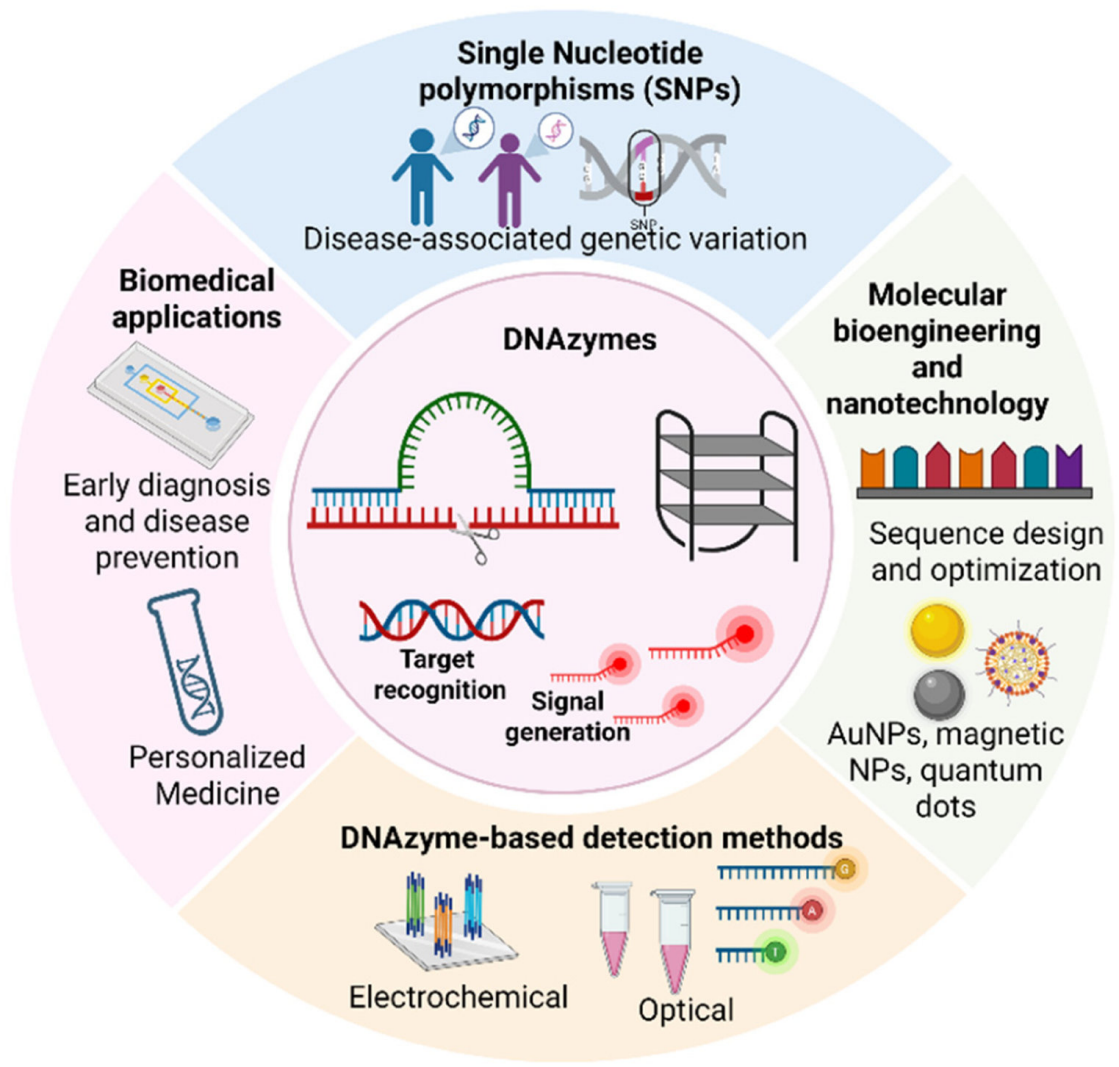
Overview of DNAzyme‐based strategies for single‐nucleotide polymorphisms (SNPs) detection and their potential biomedical applications. DNAzymes combine programmable target recognition, site‐specific catalytic cleavage, and signal generation. Their integration with molecular engineering, nanomaterials, and optical or electrochemical readouts provides a versatile framework for SNP analysis, with potential applications in early diagnosis and personalized medicine. Created with BioRender.com.

## 2 SNPs as Disease Biomarkers

SNPs are valuable biomarkers because single‐base sequence variations can influence disease susceptibility, progression, and treatment response [24]. Their widespread distribution across the genome and association with clinically relevant traits have made them useful in disease risk assessment, pharmacogenetics, and personalized medicine. The following discussion briefly highlights the biomarker value of SNPs in diabetes mellitus, cancer, and Alzheimer’s disease (AD). Beyond disease susceptibility, single‐base variations are associated with population diversity, individual traits, disease susceptibility, and differences in treatment response [25]. SNPs are the most prevalent form of DNA sequence variation and are widely used as biomarkers for disease diagnosis, risk prediction, and pharmacogenetic analysis [26, 27]. The identification of nucleic acid variations has become increasingly important for diagnosing genetic diseases, identifying genetic abnormalities, and monitoring disease and therapeutic responses [26]. However, nucleic acid targets are often present at low concentrations in biological samples, and direct sensing approaches may not always provide sufficient sensitivity and specificity. These analytical challenges have driven the development of biosensing strategies that improve nucleic acid detection performance. In this context, nanotechnology‐based bioassays and functional nucleic acid systems have emerged as promising tools for sensitive and selective SNP analysis [28].

### 2.1 Diabetes Mellitus

Type 2 diabetes mellitus (T2DM) is a metabolic disorder characterized by hyperglycemia resulting from impaired insulin secretion, insulin action, or both [29]. Its development is influenced by a combination of genetic, environmental, and lifestyle factors, with insulin resistance and pancreatic β‐cell dysfunction playing central roles [30]. SNPs in genes involved in glucose metabolism have been associated with T2DM‐related traits and susceptibility, although the strength of these associations varies among genes and populations [31]. Genetic risk scores based on combinations of these loci have also been explored for risk prediction and early diagnosis [32, 33, 34, 35]. Individual SNPs have additionally been linked to specific T2DM‐related outcomes. For example, Jarrar et al. reported a higher frequency of the CYP4F2 rs2108622 variant in patients with T2DM and an association with diabetic retinopathy [36]. Akhlaghipour et al. further highlighted the diagnostic relevance of polymorphisms in VDR, NOS, TCF7L2, and PON1 in Middle Eastern populations [37]. Together, these findings support the utility of SNPs as biomarkers of T2DM susceptibility and progression.

### 2.2 Cancer

Cancer susceptibility and progression are strongly influenced by genetic variation, and SNPs have emerged as useful biomarkers across multiple cancer types [38, 39]. Because they are common throughout the genome and can affect gene regulation or expression, SNPs are increasingly used in cancer risk assessment and biomarker discovery. Prostate cancer provides a clear example, with more than 100 common SNPs associated with increased disease risk, including variants in the 8q24 region [40]. SNP‐based susceptibility loci have also been identified in endometrial and cervical cancers [24]. In addition, Li et al. suggested that HOTAIR polymorphisms, including rs920778, rs12826786, rs874945, and rs4759314, may contribute to overall cancer susceptibility [39]. These examples illustrate the broad value of SNPs as cancer biomarkers for risk assessment and early detection.

### 2.3 AD

AD is a progressive neurodegenerative disorder and a major public health concern, particularly in aging populations [41]. SNPs linked to AD have attracted considerable interest because they can provide insight into disease susceptibility and may support earlier diagnosis [42]. The APOE ε4 allele is the strongest established common genetic risk factor for late‐onset AD [43]. SNPs in cytokine‐related genes such as IL‐1, IL‐6, IL‐10, and TNFα have also been associated with AD, supporting a role for neuroinflammatory pathways in disease development [44]. For example, Babić Leko et al. reported differences in AD susceptibility and biomarker profiles associated with TNFα‐308 genotypes [44, 45]. Overall, these findings demonstrate that SNPs can serve as informative biomarkers of AD risk and pathogenesis.

## 3 Conventional SNP Detection Methods

Accurate detection of SNPs remains analytically challenging because SNP‐containing targets differ from wild‐type (WT) sequences by only a single nucleotide [46]. Conventional methods such as PCR and RFLP have played important roles in genotyping, but they also have practical limitations that motivate the development of alternative chemical sensing platforms.

### 3.1 PCR

PCR is widely used for SNP analysis because it enables sensitive amplification of target DNA sequences [3, 47]. However, many PCR‐based workflows require enzymes, thermal cycling, multistep sample handling, and specialized instrumentation, which can limit their suitability for simple, rapid, or point‐of‐care applications [6, 48]. In addition, amplification efficiency may be affected by inhibitors present in complex biological samples [48].

### 3.2 RFLPs

RFLP analysis identifies SNPs through restriction enzyme recognition of sequence‐specific cleavage sites and has long been used in genotyping and genetic analysis [49, 50]. Although it is relatively straightforward and inexpensive, RFLP is limited by its dependence on suitable restriction sites, difficulty in multiplexed analysis, and inability to detect unknown variants efficiently. The need for carefully optimized digestion conditions and multiple processing steps can further reduce its practicality for rapid diagnostic applications [5]. These limitations have encouraged the development of more selective and adaptable approaches, including DNAzyme‐based biosensors for SNP detection.

## 4 DNAzyme: Definition and Structure

DNAzymes, or deoxyribozymes, are catalytically active single‐stranded DNA molecules capable of performing specific biochemical reactions, such as cleaving nucleic acids in the presence of metal ions, as shown in Figure 2 **.** Unlike natural enzymes, DNAzymes are typically identified through in vitro selection [51, 52, 53]. Their catalytic potential has led to applications in biosensors, therapeutic interventions, and molecular diagnostics. Structurally, as shown in Figure 2, DNAzymes generally consist of two arms that bind to the target nucleic acid, surrounding a central catalytic core that performs the reaction. The well‐characterized 10–23 and 8–17 DNAzymes rely on divalent metal ions to catalyze site‐specific cleavage of RNA‐containing substrates [51, 52, 53]. These versatile molecules adopt specific secondary and tertiary structures that are critical for their activity and can be engineered to detect various targets, making them useful in medical diagnostics and environmental monitoring [51, 52, 53].

**FIGURE 2:**
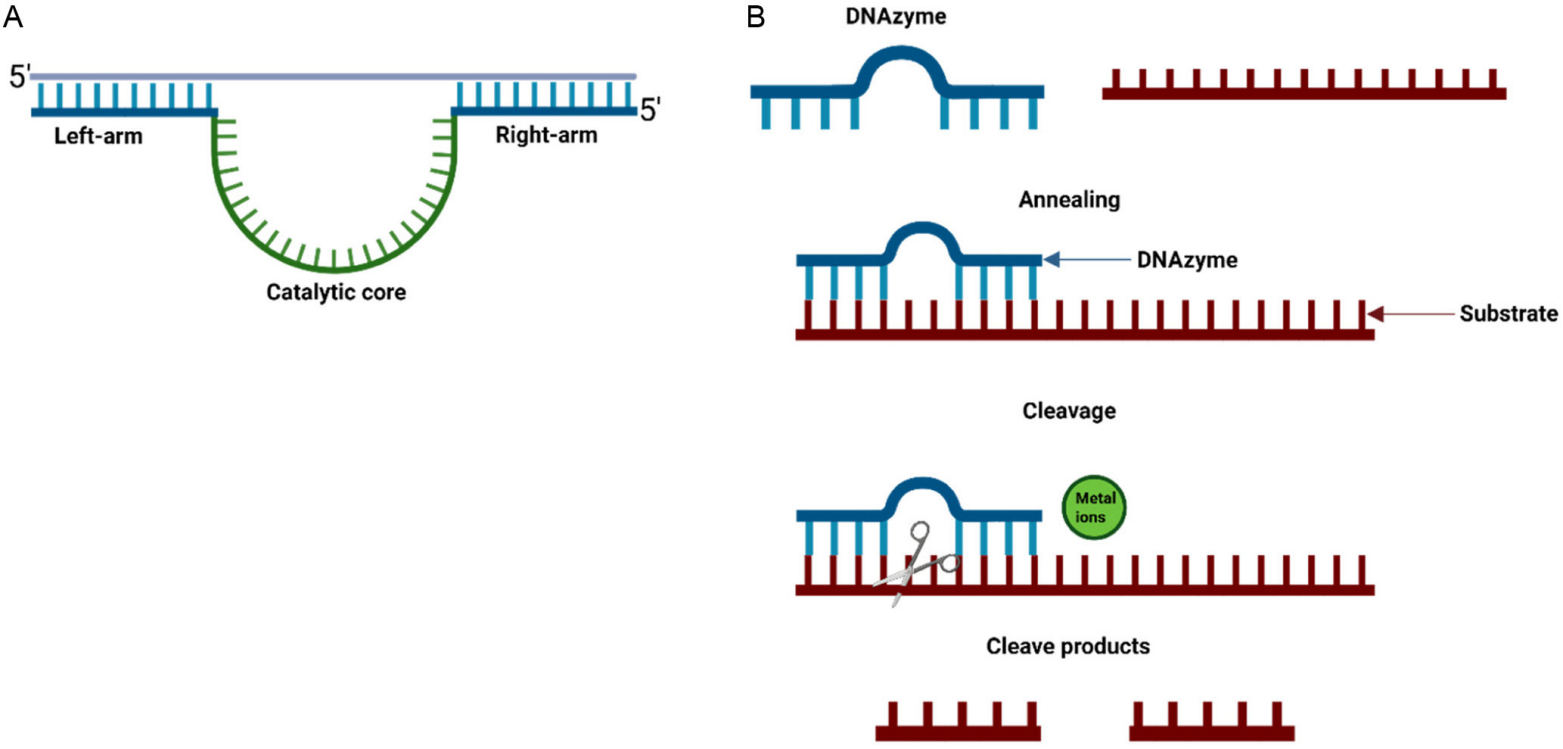
DNAzyme structure and mechanism of action. (A) Schematic representation of a DNAzyme comprising two substrate‐binding arms flanking the catalytic core. (B) DNAzyme‐mediated substrate cleavage. Upon hybridization with the substrate strand, the catalytic core cleaves the substrate in the presence of divalent metal ions (e.g., Mg^2+^ or Mn^2+^), generating cleavage products. Created with BioRender.com.

DNAzymes have garnered growing attention in the development of biosensors for detecting biomolecules and imaging applications. Their key attributes include high sensitivity, programmability, chemical modifiability, and compatibility with diverse sensing architectures [54]. Also, DNAzymes can be selected for specific targets via in vitro techniques, allowing for customization. Moreover, they can be chemically modified to incorporate functional groups tailored to their intended purpose. Additionally, DNAzymes can integrate with other functional DNA‐based systems, enhancing their ability to detect a wide array of target molecules, thanks to their programmable sequence properties [54].

Although PCR is highly effective for nucleic acid detection, many PCR‐based workflows require careful optimization, thermal cycling, specialized instrumentation, and multistep sample processing, which can limit their suitability for field‐based or point‐of‐care use [6, 48]. DNAzyme‐based sensing platforms offer an alternative that can operate under simpler conditions and may require less instrumentation [11]. A general comparison of DNAzyme‐based sensing with conventional SNP detection methods, including PCR and RFLP, is summarized in Table 1.

**TABLE 1:** Comparison between DNAzyme‐based sensing and conventional SNP detection methods [3, 4, 6, 11, 47, 48].

Feature	DNAzyme	PCR	RFLP
Speed	May range from relatively short detection steps to longer incubation times, depending on the cleavage kinetics and sensor design	Moderate (hours); requires amplification cycles	Slow; requires electrophoretic analysis
Specificity	Single‐base discrimination achievable; mismatch tolerance remains a limitation	High; risk of nonspecific amplification	High; limited to SNPs located within restriction enzyme recognition sites
Cost‐effectiveness	Potentially low reagent and instrumentation costs	Relatively higher cost	Relatively low cost
Ease of use	Potentially simple, depending on assay architecture	Complex instrumentation	Relatively simple; requires multiple steps including restriction digestion and electrophoretic analysis
Thermocycler	No need	Needs thermocycler	No need

DNAzyme‐based detection offers a compelling alternative due to its simplicity and flexibility. As catalytic nucleic acids, DNAzymes can be tailored to target and cleave specific nucleic acid sequences or detect molecules like ions. These sensors are particularly well‐suited for point‐of‐care diagnostics because they enable real‐time, single‐step detection without requiring elaborate equipment or expensive reagents, making them more practical and cost‐effective for field use [11].

## 5 Signal Transduction Methods in DNAzyme‐Based SNP Biosensors

Numerous studies have employed different DNAzymes in designing biosensors for detecting a wide range of nucleic acid‐based targets. Typically, DNAzymes serve dual roles in these systems, facilitating both molecular recognition and signal production for the biosensor [12, 55]. These biosensors rely on different signal transduction methods to convert the nucleic acid sequence recognition event into a measurable signal [56]. The detection of SNPs is closely related to nucleic acid detection, as DNAzyme‐based biosensors can be engineered to discriminate single‐base variations in target nucleic acid sequences [4, 28]. Among DNAzymes, two dominant classes have demonstrated particular promise in proof‐of‐concept biosensing systems: RNA‐cleaving DNAzymes (RCDs) and peroxidase‐mimicking DNAzymes (PMDs). RCDs facilitate the precise cleavage of an RNA dinucleotide junction, generating two fragments, while PMDs catalyze peroxidation reactions of chromophores, resulting in a detectable signal [55, 57]. Both RCDs and PMDs can be conjugated with affinity ligands, such as antibodies, to function as direct signal transducers in biosensing applications. For nucleic acid detection, DNA probes specific to target sequences can incorporate either RCD or PMD sequences to enable signal generation [20, 57]. These DNAzymes are versatile, producing a variety of signal types, including colorimetric, chemiluminescent, fluorescent, and electrochemical signals.

The following discussion will address different biosensor technologies, including electrochemical, optical, and signal amplification strategies, which are engineered by using DNAzymes and the impact they have on selectivity and sensitivity.

### 5.1 Optical‐Based Detection

Optical‐based signal transduction is one of the most common methods used in DNAzyme‐based biosensors due to its sensitivity and versatility [12]. Optical detection techniques convert molecular recognition events, such as interactions between DNAzymes and target DNA, into observable changes in optical properties like light absorption, emission, or refractive index. In DNAzyme‐based SNP biosensors, the primary optical methods used are fluorescence, colorimetric, and chemiluminescent detection, each providing sensitive readouts based on distinct optical characteristics of the DNAzyme–analyte interaction [12, 55].

Fluorescence‐based detection is a highly sensitive technique used in DNAzyme‐based SNP biosensors. In this method, fluorophores and quenchers can be attached to DNAzyme sequences or target sequences. Upon SNP recognition, a cleavage event or conformational change in the DNAzyme leads to the separation of the fluorophore from the quencher, resulting in fluorescence emission [12]. The fluorescence signal increases upon target recognition and can be used for quantitative detection under appropriately calibrated assay conditions. A schematic representation of the general fluorescence‐based DNAzyme strategy for SNP discrimination is shown in Figure 3.

**FIGURE 3:**
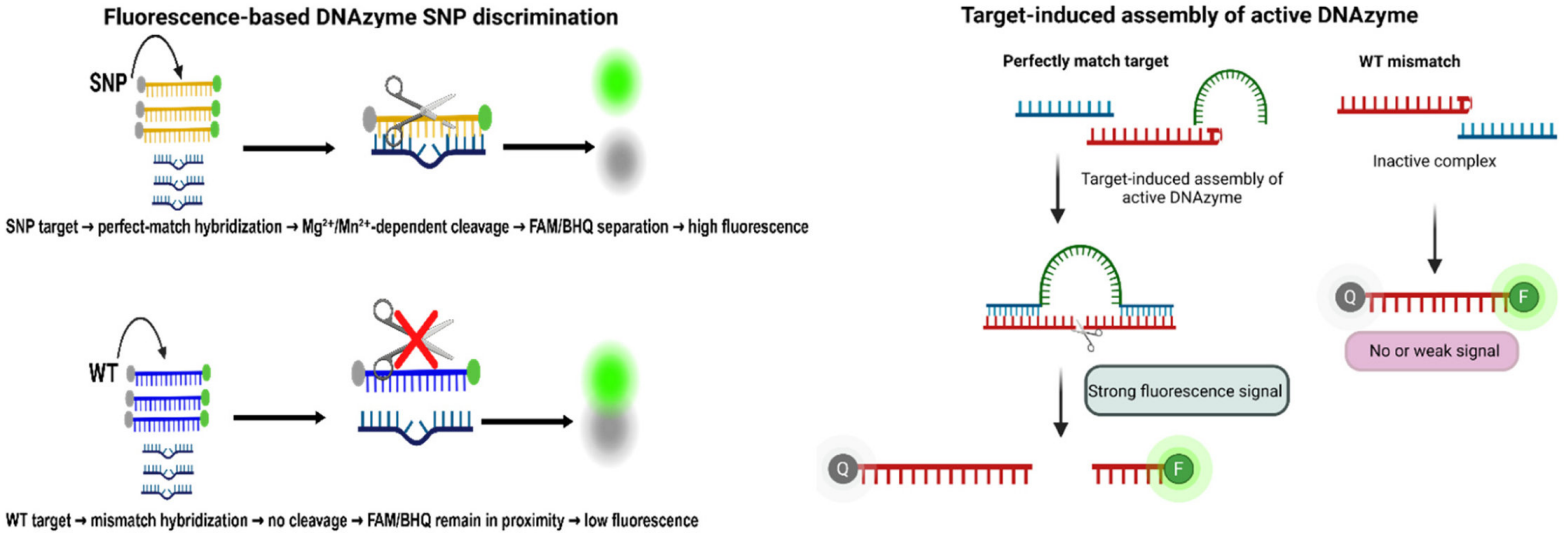
Schematic representation of two DNAzyme‐based strategies for SNP discrimination. Left: Fluorescence‐based SNP discrimination using a DNAzyme. Perfect‐match hybridization of the SNP target enables Mg^2+^/Mn^2+^‐dependent DNAzyme activity and substrate cleavage, resulting in separation of the fluorophore (FAM) and quencher (BHQ) and increased fluorescence. In contrast, mismatched hybridization with the wild‐type (WT) target suppresses DNAzyme‐mediated cleavage, maintaining FAM and BHQ in proximity and producing low fluorescence. Right: Target‐induced assembly of an active DNAzyme. A perfectly matched target promotes assembly of the active DNAzyme complex and subsequent substrate cleavage, whereas a mismatched WT target fails to support efficient DNAzyme complex formation, resulting in an inactive complex and low or no fluorescence. Created with BioRender.com.

For example, Yang et al. developed a fast and highly sensitive DNAzyme‐based detection platform called REVEALR (RNA‐encoded viral nucleic acid analytic reporter) for identifying SARS‐CoV‐2 variants. The REVEALR system correctly identified the tested SARS‐CoV‐2 variants in 34 sequence‐verified clinical samples with 100% accuracy [58]. They introduced an enhanced version of the REVEALR platform, termed digital droplet REVEALR (ddREVEALR), designed for direct viral detection and precise sample quantitation [59]. The ddREVEALR platform leverages a signal amplification strategy incorporating chemical modifications, DNAzyme multiplexing, and volume compression for heightened sensitivity. Utilizing an AI‐assisted, image‐based readout, ddREVEALR demonstrated a 95% positive predictive agreement in an evaluation of 20 nasopharyngeal swab samples [59].

In terms of nucleic acid detection, a fluorescence‐based DNAzyme biosensor was designed for highly sensitive detection of miRNAs [60]. The biosensor operates by utilizing a DNAzyme that recognizes Mg^2+^ and catalyzes a self‐cleavage reaction, leading to cyclic amplification of the fluorescence signal. Upon interaction with the target miRNA, the DNAzyme induces a conformational change that triggers the fluorescence signal, allowing for real‐time and quantitative detection. The biosensor exhibits a detection limit (LOD) as low as 0.49 pmol/L, demonstrating excellent sensitivity [60].

Another study presents a novel, protein‐ and enzyme‐free nanoconstruction, termed the four‐armed DNA machine (4DNM), which eliminates the need for both amplification and high‐temperature annealing in the detection of SARS‐CoV‐2 [61]. The 4DNM utilizes a binary deoxyribozyme sensor integrated within a DNA nanostructure, equipped with four RNA‐binding arms. The additional arms were shown to improve the LOD by at least 10‐fold. This sensor successfully distinguished SARS‐CoV‐2 from other respiratory viruses and accurately identified five positives and six negative clinical samples, as verified by quantitative RT‐PCR [61]. The same research group introduced an innovative DNA machine biosensor, incorporating an F‐sub‐1 cleavage with a DNA structure that has four analyte‐binding arms, substantially enhancing sensitivity for synthetic DNA analyte detection [62]. Compared with the hookless system, HDNM provided an approximately 80‐fold improvement in DNA detection and a 13‐fold improvement in RNA detection while reducing incubation time from 3 to 1 h [62].

In 2026, Ace et al. designed a 10–23 binary DNAzyme‐based sensor that was able to differentiate the challenging G → A substitution with a differentiation factor of >98% [63]. Substitutions G → A are very frequent SNVs among multiple cancer types, including lung, breast, and skin cancers. These substitutions are particularly challenging to discriminate because of the small free‐energy difference between fully matched and mismatched probe/analyte complexes, including the structurally similar purine–purine substitutions [63]. This results in only minor duplex destabilization, which leads to minimal changes in hybridization thermodynamics. Therefore, the authors designed a binary DNAzyme composed of two strands, Dza and Dzb, which hybridize with neighboring positions of the analyte and assemble into an Mg^2+^ dependent catalytic core that cleaves a reporter substrate to generate an optical signal [63]. The authors mentioned that the differentiation factor of their sensor design increases from >83% to >98% after optimization of the SNV position. The authors found that positioning the G → A substitution close to the DNAzyme catalytic core introduced an additional SNV discrimination mechanism that was not governed solely by duplex hybridization thermodynamics [63]. Representative DNAzyme‐based nucleic acid biosensors, including their target, limit of detection, and reported selectivity, are summarized in Table 2.

**TABLE 2:** Representative DNAzyme‐based nucleic acid biosensors and their relevance to SNP/SNV detection.

Biosensor design	Target	LOD	Selectivity	References
REVEALR split DNAzyme, competitive binding	SARS‐COV‐2 Variants	10 copies/µL	The biosensor can genotype SARS‐CoV‐2 variants by recognizing base‐pair mismatches in a competitive‐binding format	[58]
Target‐induced rolling‐circle amplification complemented with self‐cleaving DNAzyme and fluorescence release	miRNA 21	0.49 pmoL L^−1^	Strong discrimination against nontarget RNAs, including mut‐miR‐21 and miR‐16, off‐target fluorescence remained close to the DNAzyme background	[60]
Fluorescence‐based hook‐equipped four‐armed DNA nanomachine	SARS‐CoV‐2	0.025 pM	The biosensor is highly selective, as it did not produce significant fluorescence even when incubated with high concentrations of the nonspecific Influenza A virus for 3 h at 55 °C	[62]
10–23 binary DNAzyme with core‐adjacent SNV recognition	G → A single‐nucleotide substitution	Not reported	Positioning the SNV adjacent to the catalytic core increased the differentiation factor of the target to >98%	[63]

Colorimetric detection offers a simple and visual way to detect DNA, especially SNPs, without the need for sophisticated equipment. In this regard, a novel DNAzyme‐based biosensor employing a three‐way junction (TWJ) DNAzyme probe has been developed for the colorimetric detection of SNPs [64]. The sensor utilizes a DNAzyme‐linked hairpin DNA as the signal probe. In the presence of the target DNA, the signal probe, assistant probe, and target DNA hybridize to form a TWJ, which dehybridizes the DNAzyme sequence. Once the DNAzyme sequence is exposed, it forms a G‐quadruplex(G4)‐hemin complex in the presence of hemin, catalyzing the oxidation of colorless 2,2′‐azinobis(3‐ethylbenzothiozoline)‐6‐sulfonic acid (ABTS) by hydrogen peroxide, resulting in a distinct color change that can be observed visually. This sensor demonstrates high specificity for SNP detection and can detect target DNA at concentrations as low as 0.25 nmol/L [64].

### 5.2 Electrochemical‐Based Detection

Electrochemical DNAzyme detection of DNA involves using DNAzymes as signal‐generating components that catalyze redox reactions on an electrode surface, enabling sensitive and specific DNA analysis through measurable electrochemical signals. Most of the DNAzymes used to develop electrochemical biosensors are either RCD or PMD. G4‐forming DNAzyme PMDs catalyze H_2_O_2_‐mediated oxidation of electron‐rich molecules, mimicking the activity of peroxidase enzymes. This property makes PMDs effective reporter systems for electrochemical detection [65]. In PMD electrochemical biosensor design, fully functional PMDs are typically formed by assembling one or more G‐rich sequences that are initially inactive, enabling sensitive detection through electrochemical signals generated by the catalysis of H_2_O_2_ [65]. In this regard, a hemin/G4 horseradish PMD is assembled on Au electrodes, catalyzing the reduction of H_2_O_2_ and providing bioelectrocatalytic activity [66]. This DNAzyme‐based configuration enables electrochemical sensing of glucose oxidase (GOx) activity, DNA, and low‐molecular‐weight targets like adenosine monophosphate (AMP). Hairpin structures containing both a G4 sequence and a complementary sequence for the target open in the presence of analytes, activating the DNAzyme. The bioelectrocatalytic currents from H_2_O_2_ reduction yield quantitative signals, achieving LOD of 1 pM for DNA and 1 µM for AMP [66].

The RNA detection by using electrochemical PMD and RCD platforms has been developed. Meng et al. developed a signal‐on biosensor using AuNPs conjugated with Mn^2+^‐specific RCDs on a PMD‐functionalized Au electrode with methylene blue (MB) for signal amplification [67]. The RCD on the AuNP binds a complementary DNA sequence that initially blocks substrate access. Upon miRNA (miR‐486‐5p) interaction, RCD moves to the substrate strand, where Mn^2^^+^ mediates RNA cleavage, releasing output DNA. This output initiates a HCR on the gold electrode, forming PMD with MB for electrochemical signaling, achieving a LOD of 5.68 fM in buffer [67].

Xue et al. combined DNAzyme walkers with strand displacement reactions for highly sensitive electrochemical detection of miRNAs [68]. A transduction hairpin selectively binds miR‐155, forming an initiator duplex that interacts with three hairpins through a toehold‐mediated strand displacement reaction, generating a three‐legged DNAzyme walker [68]. When activated by Mg^2+^, the DNAzyme cleaves a substrate hairpin immobilized on a gold electrode and intercalated with MB, producing a signal proportional to target concentration. This approach demonstrated an LOD of 0.27 fM, though the 2‐h reaction time remains as a limitation [68]. Another example of electrochemical DNAzyme detection of DNA is the use of AuNPs as carriers for hemin/G4 DNAzyme units that are conjugated with a DNA sequence complementary to the target DNA [69].

Enzymes like GOx and glucose dehydrogenase are linked to electrodes via the reconstitution of their apo‐proteins on AuNPs functionalized with cofactors like flavin adenine dinucleotide or pyrroloquinoline quinone. Similarly, AuNPs integrated into polyaniline microrods provide an optimal matrix for GOx electrode contact. AuNPs can also carry hemin/G4 DNAzyme units with a DNA detection segment, enabling electrochemical signal transduction for DNA analysis [69]. While existing electrochemical DNAzyme detection strategies were not initially developed specifically for SNP analysis, they hold significant potential for SNP detection due to their target‐specific recognition and catalytic capabilities. Adapting these methods by incorporating RCDs and PMDs on conductive platforms, such as AuNP‐functionalized electrodes, could enable the precise recognition of single‐nucleotide variations. Despite this potential, electrochemical DNAzyme‐based SNP detection remains an underexplored area, presenting opportunities for further research and development in this field.

## 6 Signal Amplification Strategies

Signal amplification strategies for DNAzyme‐based biosensors can be broadly classified into two main types: direct response amplification strategies and combinational response amplification strategies [20]. Direct response amplification strategies couple DNAzyme activity to a single amplification module, including approaches based on strand displacement amplification (SDA), loop‐mediated isothermal amplification (LAMP), RCA, and HCR [20]. In contrast, combinational response amplification strategies integrate DNAzyme activity with two or more sequential or coupled amplification mechanisms to further enhance signal output [20, 57]. These include techniques such as cascade strand displacement coupled with G4 peroxidase activity, dual‐isothermal amplification paired with enzymatic signal enhancement or SDA, and binary deoxyribozyme sensors with PCR‐like cycling for colorimetric detection [20]. Each method leverages distinct features to enhance signal generation and analytical sensitivity; when coupled with allele‐specific recognition, these strategies can also support SNP discrimination [20, 57].

LAMP is a highly efficient nucleic acid amplification technique widely used for rapid and sensitive detection of specific DNA or RNA sequences. To simplify and enhance the reliability of LAMP detection, a cascade signal‐on colorimetric DNAzyme LAMP (dLAMP) sensor was developed [70]. This sensor integrates LAMP’s amplification capability with DNAzyme catalytic activity for ultrasensitive target detection. A signal inner primer containing a 17‐nt DNAzyme complementary sequence, acting as a signal precursor, was incorporated for sequential amplification and colorimetric readout [70]. In the presence of target DNA, LAMP selectively amplifies the signal precursor, generating numerous DNAzyme sequences. Upon hemin addition, free DNAzyme fragments were released through ultrasonication, and pPNA form G4‐hemin conjugates, providing direct colorimetric signals. This method achieved an LOD of 0.5 pg genomic DNA in a single step, without gels or specialized equipment [70]. As a combinational response amplification, the performance of the G4‐based SNP assay can be further enhanced by strengthening signal amplification through a dual‐isothermal SDA strategy [71]. Allele discrimination was achieved by combining discrimination probes 1 and 2 with a common probe 3 and a ligase. SDA began with the end of probe 3 as a primer, and the resulting product was combined with probe 4 for a second amplification round. Upon adding hemin, the amplicons formed a peroxidase‐like DNAzyme, initiating a chemiluminescence reaction between luminol and hydrogen peroxide. This approach yields superior selectivity with a signal‐to‐background ratio of 150:1, surpassing other G4‐based SNP assays [71].

Nonenzymatic isothermal amplification methods, including DNAzyme‐catalyzed reactions, CHA, and HCR, offer simple operation, high amplification efficiency, and enhanced target detection sensitivity, making them widely utilized in DNAzyme‐based nucleic acid sensing [20, 72]. Combining isothermal amplification strategies with DNAzyme‐catalyzed reactions in a cascade design significantly enhances the detection sensitivity of DNAzyme‐based assays [20]. Among enzyme‐free systems, CHA operates as an entropy‐driven, isothermal reaction where an initiator cross‐opens two DNA hairpins to produce stable double‐stranded DNA (dsDNA) with a 50–100‐fold signal increase within hours. Its simple design and low background make CHA ideal for detecting nucleic acids [73]. HCR, also enzyme‐free and isothermal, forms linear DNA nanostructures via cross‐opening and self‐assembly of dsDNA, enabling high amplification with minimal complexity [74]. In this regard, a versatile signal amplification platform was developed based on an isothermal concatenated CHA–HCR–DNAzyme strategy for the sensitive detection of nucleic acids [75]. In this approach, the upstream CHA stage recognizes a specific initiator, generating multiple dsDNA strands with embedded HCR trigger sequences that activate the cross‐opening of downstream hairpin reactants in the HCR process. Long dsDNA copolymers are subsequently produced, encoding tandem DNAzymes that cleave fluorophore‐labeled substrates to produce an amplified fluorescence signal. Through successive amplification cascades, this CHA–HCR–DNAzyme system effectively transduces the target analyte into a highly amplified readout [75]. RCA, in contrast, uses DNA polymerases like phi29 in an enzymatic process to extend primers along circular templates, producing long single‐stranded DNA with repeated sequences [20, 76]. Zhou et al. demonstrated that multienzymatic systems, in combination with a novel RCA technique, yield an effective approach for amplifying super‐long dsDNA [77]. DNA circles were selected to span a wide sequence range (2–160 kb), enhancing the method’s applicability and generalizability. Leveraging this amplified super‐long dsDNA, a triple‐enzyme correction system was developed to repair structural abnormalities, improving reproducibility.

This technique enables target‐specific amplification of super‐long dsDNA from circular templates, extending amplification capabilities and advancing DNA amplification technology [77]. The amplification strategies summarized in Table 3, while largely centered on nucleic acid detection, provide foundational insights relevant to the precise detection of SNPs within DNAzyme‐based biosensors. Direct amplification methods such as LAMP, RCA, and HCR, along with combinational approaches such as CHA–HCR–DNAzyme cascades, can enhance signal generation and analytical sensitivity, providing useful design principles for SNP‐detection platforms. Techniques like enzyme‐free CHA and HCR amplify target signals with minimal complexity, while enzymatic approaches, including RCA with multienzyme systems for super‐long dsDNA amplification, expand the range and robustness of DNA amplification and may provide useful design principles for downstream SNP assay development. Together, these strategies illustrate versatile approaches for enhancing signal generation and analytical sensitivity in DNAzyme‐based nucleic acid assays and may inform the further development of sensitive SNP‐detection platforms.

**TABLE 3:** Direct and combinational signal amplification strategies used in DNAzyme‐based nucleic acid biosensors, with relevance to SNP detection.

Amplification strategy	Type	Amplification mechanism	Key features/advantages	Detection limit (LOD)	References/example
LAMP (Loop‐mediated isothermal amplification)	Direct response	Target DNA triggers loop‐mediated amplification; signal inner primer encodes DNAzyme sequence; hemin forms G4‐hemin conjugates for colorimetric readout	One‐step, rapid, enzyme‐based colorimetric output; no gels or specialized equipment	0.5 pg genomic DNA	[70]
Dual‐isothermal strand displacement amplification (SDA)	Combinational response	Discrimination probes initiate SDA with ligase; product triggers second SDA; hemin produces chemiluminescent signal	Dual amplification; high selectivity (signal/background = 150:1); enhanced G4‐DNAzyme activity	0.1 fM	[71]
CHA (Catalytic hairpin assembly)	Direct (enzyme‐free)	Entropy‐driven hairpin opening reaction forming dsDNA products	Enzyme‐free, low background noise, ~50–100× signal increase	Not specified	[73]
HCR (Hybridization chain reaction)	Direct (enzyme‐free)	Sequential hybridization of DNA hairpins forming long linear dsDNA	Simple, isothermal, generates strong fluorescence or colorimetric output	Not specified	[74]
Concatenated CHA–HCR–DNAzyme system	Combinational response	CHA generates HCR trigger sequences; HCR produces long dsDNA containing tandem DNAzymes; cleavage of fluorophore‐labeled substrate amplifies signal	Highly cascaded amplification; ultrasensitive; enzyme‐free	Not specified	[75]
RCA (Rolling circle amplification)	Direct enzymatic	DNA polymerase (e.g., phi29) extends primer along circular DNA templates, producing long ssDNA repeats	High yield, simple, isothermal, compatible with DNAzyme signaling	Not specified	[20, 76]
Multienzyme RCA (super‐long dsDNA amplification)	Combinational enzymatic	Multienzyme correction system amplifies circular templates (2–160 kb) to form super‐long dsDNA; structural correction enhances reproducibility	Expands the range and robustness of DNA amplification; potentially useful for downstream SNP assay development	Not specified	[77]

## 7 DNAzymes for the Detection of SNPs

SNPs can contribute to the genetic basis of many diseases, including cancer and cardiovascular disease [78, 79, 80]. SNP analysis also plays an important role in personalized medicine [26, 81]. Thus, engineering platforms that can detect SNPs with high sensitivity and selectivity are of utmost importance in diagnosing diseases in their early stages, which can enhance patient survival rates in many diseases. The need for new SNP‐detection methods has encouraged the development of technologies that provide improved sensitivity, selectivity, and potentially lower cost, including DNAzyme‐based sensors.

Compared with alternative SNP detection methods, DNAzyme‐based sensors can be advantageous when target recognition can be directly coupled to catalytic activation and repeated signal generation. In appropriately designed systems, correct target hybridization enables DNAzyme assembly or activation, whereas a single‐base mismatch can destabilize the recognition complex and suppress the catalytic activity. Once activated, the DNAzyme may catalyze multiple substrate conversion events, providing repeated signal generation rather than a single stoichiometric recognition event. This feature can be particularly useful when enhanced signal output is required without additional target amplification. In fact, the choice between DNAzymes and other SNP detection methods should depend on the required sensitivity, target characteristics, reaction conditions, and desired readout mechanism. The following discussion will explain the different optical techniques for the detection of SNPs via DNAzyme‐based biosensors.

Zhang et al. designed a highly selective and sensitive optical biosensor based on DNAzyme activity for the detection and discrimination of SNV [82]. The success of this biosensor is attributed to the discrimination property of endonuclease IV (Endo IV) and the different hydrolysis behavior between free DNAzyme and DNAzyme conjugated to AuNPs [82]. The recognition of mutant‐type target by Endo IV releases free DNAzyme from the probe, which subsequently activates a DNAzyme motor with the assistance of cofactor Mn^2+^, generating an amplified fluorescence signal [82]. In the absence of the mutant‐type target, the DNAzyme motor is not activated, and no amplified fluorescence signal is generated. The assay was able to discriminate five clinically relevant SNVs at mutant allele fractions ranging from 0.01% to 0.05% in the presence of WT target [82].

In another study, a protein enzyme‐free biosensor was designed to detect SNPs in a selective and sensitive manner via the utilization of an asymmetric multiarm fluorescent MNAzyme system [83]. The system is composed of two enzyme chains (E1 and E2) and a substrate (S) labeled with a fluorophore and a quencher [83]. The two enzyme chains are composed of three regions, as follows: (I) a recognition arm, (II) a catalytic core, and (III) a substrate arm [83]. This biosensor was used to detect EGFR T790M (c.2369C > *T*) gene as a mutant‐type target [83]. Upon exposure to the mutant‐type target, the two recognition arms (E1 and E2) hybridize with it, activating the MNAzyme to cleave the substrate, which contains a fluorophore and a quencher, thereby producing a fluorescence signal [83]. After the cleavage of the substrate, the MNAzyme simultaneously leaves the cleaved substrate to start a new cycle. This mechanism leads to the generation of an amplified fluorescence signal. In contrast, when applying this biosensor to the corresponding WT EGFR sequence, the MNAzyme was not activated because the recognition arms of E1 and E2 could not hybridize with the WT due to a base mismatch [83]. The authors reported a concentration‐based LOD of 0.59 nM for the EGFR T790M mutant target and were able to discriminate a mutant allele fraction as low as 0.83% in the presence of the corresponding WT sequence [83].

In 2022, Li et al. designed an optical DNAzyme‐based biosensor to detect SNPs of the K‐ras gene [84]. The mechanism of this biosensor can be explained in two scenarios, as follows: (I) When the biosensor is exposed to the WT of the K‐ras gene, it hybridizes with two oligonucleotides referred to as probe oligonucleotide and invader oligonucleotide to form a trinucleotide repeat structure [84]. This trinucleotide repeat is recognized by an enzyme known as flap endonuclease 1 (FEN1), which results in the cleavage of the 5′ flap [84]. The cleavage of the trinucleotide repeat inhibits the interaction between this trinucleotide repeat structure and what they call the supporting strand (S), which in return prevents the initiation of the subsequent catalytic hairpin association (CHA) [84]. (II) When the biosensor is exposed to the mutant‐type of K‐ras gene, it forms a nontrinucleotide repeat (NTR) substrate [84]. The NTR prevents it from recognition and cleavage by FEN1 and triggers the activity of the subsequent CHA of three hairpins for the creation of tripartite DNAzyme junctions [84]. These DNAzymes are responsible for cleaving the substrate and separating the fluorophore from the quencher, thereby producing the fluorescence signal. The authors stated that using their biosensor design, they were able to detect mutant forms of the K‐ras gene at 4.23 fM [84].

Many other studies used DNAzyme for the detection of SNPs to produce optical signals, such as chemiluminescence. This is due to the fact that DNAzymes are very stable and can be easily synthesized over native enzymatic labels. A study designed a simple enzyme‐free and label‐free DNAzyme sensor for the detection of rs242557 SNP genotyping [85]. The rs242557 SNP is known for its association with AD [85]. The DNAzyme sensor works as follows: (I) In the absence of the rs242557 SNP, the DNA hairpins and the partially complementary nucleotide sequences will not open their loops for hybridization, and this is due to the thermodynamic barriers [85]. (II) In the presence of the target DNA, the hairpins are opened through target‐triggered hybridization, promoting DNAzyme assembly and subsequent chemiluminescence generation [85].

The colorimetric detection method is also widely used in the detection of SNPs using DNAzyme‐based biosensors. This is due to the simplicity, accuracy, and cost‐effectiveness of colorimetric methods [86]. Several researchers have integrated both technologies to combine the advantages of the colorimetric methods mentioned above with the high selectivity and sensitivity of DNAzyme for detecting SNPs. For example, Ouedraogo et al. designed a sensitive and selective colorimetric assay for the detection of SNPs using the combination of RCA and G‐quadruplex/hemin DNAzyme [87]. First, a padlock probe (PLP) DNA that has the potential to differentiate a single base mismatch hybridizes with ssDNA [87]. Subsequently, an RCA event occurs to form an abundance of G‐quadruplexes (GQ‐RCA), which are converted to a DNAzyme (G‐quadruplex/hemin complex) once hemin is added [87].

The G4/hemin DNAzymecatalyzes H_2_O_2_‐mediated oxidation of colorless TMB to coloredoxTMB, providing the colorimetric readout [87]. The authors mentioned that this assay has a LOD of 2.14 pM. Moreover, they mentioned that they were able to detect mutations from samples obtained from breast cancer patients with 100% agreement with Sanger sequencing in the tested samples [87]. In another study, SNPs were detected by using the combination of G‐quadruplex and hemin binding [88]. In fact, this combination provides the ability for the detection of SNPs in a highly sensitive manner and without the need for high‐cost instruments to detect the color changes since the color change produced from this method can be visible to the naked eye at room temperature [88]. The authors stated that they designed a new split mode to divide hemin‐binding DNA aptamer into two parts: one has three *GGG* repeats, and the other part has one *GGG* repeat, the 3:1 split mode. They also mentioned that using the G‐quadruplex‐containing peroxidase DNAzyme utilizes the 3:1 split mode and were able to detect the SNP with a LOD in the nM range [88].

As discussed above, the variation in LODs among DNAzyme‐based SNP assays reflects not only differences in DNAzyme sequence and catalytic efficiency but also differences in the overall assay architecture. For example, the MNAzyme‐based EGFR T790M assay achieved a LOD in the nanomolar range through target‐dependent assembly, followed by catalytic substrate cleavage, whereas the K‐ras assay reached the femtomolar range by integrating FEN1‐mediated target discrimination with CHA and subsequent DNAzyme formation. In fact, coupling DNAzyme catalysis with upstream target amplification or downstream signal amplification strategies can improve analytical sensitivity. Likewise, electrochemical or colorimetric transduction can enhance signal readability, particularly when combined with catalytic or nanomaterial‐based amplification. Moreover, intrinsic factors such as DNAzyme sequence, catalytic turnover rate, recognition‐arm design, target accessibility, mismatch position, ionic conditions, and target secondary structure can influence both sensitivity and reaction kinetics. Therefore, the LOD and assay time of DNAzyme‐based biosensors are highly dependent on both the intrinsic properties of the DNAzyme and the overall sensor design.

Overall, the tunable catalytic and recognition properties of DNAzymes, together with the biomarker potential of disease‐associated SNPs, support the continued development of DNAzyme‐based platforms for SNP screening and molecular diagnostics. These approaches may complement conventional methods such as PCR by offering simpler and potentially lower‐cost assay formats.

## 8 Efforts to Improve the Sensitivity and Specificity of DNAzyme‐Based SNP Detection

### 8.1 Binding Arms Optimization

The binding arms of a DNAzyme, which flank its catalytic core, play a pivotal role in its interaction with target sequences, including those containing SNPs. These arms are designed to hybridize with complementary regions of the substrate, ensuring stable and specific binding. Binding‐arm length is particularly important because excessively short arms may not provide sufficient hybridization stability, whereas overly long arms may increase the likelihood of nonspecific interactions with unintended sequences. The nucleotide sequences of the binding arms must also be carefully optimized to complement the target region while minimizing mismatched hybridization. These considerations are especially critical for SNP detection, where sequences differing by only a single nucleotide must be distinguished.

The position of a mismatch within the binding arm can strongly influence DNAzyme activity and SNP discrimination. Mismatches located near the cleavage site may produce a greater reduction in catalytic activity because they can disrupt both DNAzyme–substrate hybridization and the local geometry required for efficient cleavage. In contrast, mismatches farther from the cleavage site may be better tolerated, particularly when sufficient duplex stability is maintained. Binding arm length also affects SNP discrimination. Shortening the arms can improve specificity because a single‐base mismatch generally has a greater destabilizing effect on shorter duplexes, making hybridization with mismatched sequences thermodynamically less favorable [10]. However, excessively short arms may compromise binding efficiency and reduce catalytic activity. Therefore, careful optimization of binding‐arm length, nucleotide sequence, and mismatch position is essential for balancing sensitivity and specificity in DNAzyme‐based SNP detection assays [89, 90].

### 8.2 Cofactor Optimization

DNAzymes rely on specific metal ions, known as cofactors, to facilitate their catalytic activity. These metal ions play a dual role: stabilizing the DNAzyme’s structure and participating in the cleavage reaction. The choice of cofactor can profoundly influence both the activity and specificity of a DNAzyme, particularly in SNP detection assays where a single‐nucleotide difference must be discriminated. Commonly used cofactors include magnesium ions (Mg^2+^), which are widely utilized due to their availability and stabilizing properties, as well as zinc (Zn^2+^) and manganese (Mn^2+^) ions, which can enhance the catalytic efficiency of certain DNAzymes by interacting more directly with the substrate [89, 91].

Different metal ions can exert distinct effects on DNAzyme folding and catalytic activity. Consequently, both cofactor identity and concentration should be optimized for each DNAzyme architecture [89, 91]. In SNP detection, the metal cofactor’s role becomes even more critical because subtle structural differences between the WT and mutant alleles may influence the binding and cleavage efficiency. By tailoring the cofactor composition and concentration, researchers can achieve a higher level of precision, ensuring that the DNAzyme selectively cleaves the intended target sequence while ignoring mismatched ones. Because cofactor conditions influence DNAzyme structure and catalytic efficiency, they may also affect discrimination between matched and mismatched substrates. Cofactor optimization is therefore an important consideration in SNP assay design [89, 90, 91].

### 8.3 Reaction Conditions

The reaction conditions, particularly temperature and ionic strength, are pivotal in achieving efficient hybridization and catalytic activity in DNAzyme‐based SNP detection assays. The annealing temperature must be carefully optimized to allow stable hybridization of the DNAzyme with its substrate while preventing nonspecific interactions with mismatched sequences. Reaction temperature can strongly influence DNAzyme–substrate hybridization, catalytic activity, and sensor response; therefore, it should be optimized for each DNAzyme and target architecture [92].

Ionic strength, controlled by salts like NaCl or KCl, also influences the hybridization and folding of DNAzymes. These ions stabilize the negatively charged phosphate backbone of the DNA, ensuring proper folding of the DNAzyme and substrate complexes. However, excessive ionic strength can shield mismatches, leading to reduced specificity. Adjusting these parameters based on the specific DNAzyme and substrate sequence ensures the highest sensitivity and specificity for SNP detection [89, 91].

In addition, reaction temperature directly affects DNAzyme catalytic activity, and the optimal temperature depends on the specific DNAzyme architecture and substrate [92]. Precise control over these conditions ensures not only robust hybridization but also efficient cleavage of the target SNP sequence, providing reliable results in biosensing applications.

### 8.4 Assay Design

Assay design plays a crucial role in determining the overall sensitivity and specificity of DNAzyme‐based SNP detection systems. Incorporating amplification strategies, such as RCA, can dramatically enhance the sensitivity of these assays. RCA works by amplifying the signal from a single binding event, generating a large number of DNAzyme molecules or reaction products from a single template. This ensures that even low‐abundance SNPs can be detected with high accuracy [87].

Additionally, employing quenched fluorescent substrates labeled with fluorophores (e.g., FAM) and quenchers (e.g., BHQ) allows real‐time monitoring of DNAzyme activity. Upon cleavage of the substrate, the fluorophore is released from the quencher, producing a measurable fluorescence signal that correlates with DNAzyme activity. This approach enhances the specificity of the assay as fluorescence is only generated when the target sequence is cleaved [28].

Multivalent probes, which present multiple DNAzyme‐binding sites, further improve the signal‐to‐noise ratio by increasing the likelihood of DNAzyme interaction with the target SNP. By integrating these advanced design elements, researchers can achieve both high sensitivity for detecting rare SNPs and high specificity to minimize false positives, including in some biological samples under optimized conditions [28]. The performance of DNAzymes in complex biological matrices remains challenging as nucleases, proteins, ionic components, nonspecific interactions, and other matrix constituents can interfere with DNAzyme stability, target recognition, catalytic activity, and signal transduction. Therefore, successful application in biological samples generally requires additional optimization of the sensing architecture and assay conditions.

### 8.5 Structural and Thermodynamic Factors

The structural and thermodynamic properties of the DNAzyme–substrate complex are critical in ensuring high specificity in SNP detection. One of the key parameters is the Gibbs free energy (Δ*G*), which determines the stability of the complex. A lower Δ*G* indicates a more stable interaction between the DNAzyme and its target, enabling strong hybridization and efficient cleavage. However, excessive stability can also lead to reduced discrimination between target and nontarget sequences, necessitating careful optimization of thermodynamic parameters [89, 93].

The presence of secondary structures in the DNAzyme or substrate can interfere with hybridization, further affecting specificity. For example, hairpins or loops in the substrate might prevent DNAzyme from accessing the cleavage site [93]. Additionally, incorporating mismatches in the binding arms of the DNAzyme can destabilize nonspecific interactions, improving SNP discrimination [90, 93]. These thermodynamic considerations are essential for designing DNAzyme systems that are both robust and highly selective.

### 8.6 Computational and In Silico Design

Computational tools and in silico simulations have become invaluable in the design of DNAzymes for SNP detection. These tools enable researchers to predict the interactions between DNAzymes and their substrates, allowing for the rational design of highly specific and efficient systems. By modeling the thermodynamic and kinetic properties of the DNAzyme–substrate complex, these tools help identify optimal sequences and configurations that maximize specificity while maintaining high catalytic activity [4].

One powerful approach involves simulating toehold‐mediated strand displacement reactions, where a short single‐stranded region of the DNAzyme initiates binding with the target substrate. These simulations can predict how mismatches near the SNP site affect binding and cleavage efficiency, enabling the design of DNAzymes that are highly sensitive to single‐nucleotide differences [94]. Furthermore, computational methods can evaluate the stability of secondary structures and predict the folding dynamics of the DNAzyme, ensuring that the active site remains accessible for catalysis. These advancements significantly reduce the time and effort required for experimental optimization, making computational design a cornerstone of modern DNAzyme‐based SNP detection strategies [4, 95].

### 8.7 Experimental Validation

Experimental validation is a crucial step in ensuring the sensitivity and specificity of DNAzyme‐based SNP detection systems. This process involves testing the DNAzyme against both the target sequence (e.g., the SNP‐containing allele) and closely related nontarget sequences (e.g., the WT allele). By systematically comparing the cleavage efficiency and binding affinity for each sequence, researchers can confirm the DNAzyme’s ability to distinguish single‐nucleotide differences with high accuracy [90, 96].

Validation also includes optimizing reaction conditions, such as temperature, ionic strength, and cofactor concentrations, to minimize nonspecific interactions. Control experiments, where either DNAzyme or substrate are omitted, are essential for identifying background signals and ensuring that observed activity is specific to the DNAzyme–substrate interaction. Additionally, using advanced detection methods, such as fluorescence‐based assays or gel electrophoresis, provides quantitative and qualitative insights into DNAzyme performance [93]. These validation steps are critical for translating DNAzyme‐based assays from the laboratory to real‐world applications, ensuring their reliability and robustness in diverse diagnostic settings. The major strategies discussed above for improving the sensitivity and specificity of DNAzyme‐based SNP detection are summarized in Table 4.

**TABLE 4:** Strategies employed to improve the sensitivity and specificity of DNAzyme‐based SNP detection. Optimizing structural design, reaction environment, and computational prescreening collectively enhance single‐nucleotide discrimination and robustness of DNAzyme assays.

Optimization strategy	Mechanistic focus	Effect on sensitivity	Effect on specificity	Key parameters/examples	References
Binding arm optimization	Adjusts hybridization strength between the DNAzyme and target substrate	Improves hybridization efficiency, enhancing cleavage rate	Increases discrimination by minimizing mismatched binding	Arm length and sequence should be optimized; mismatch position can strongly influence cleavage	[89, 90]
Cofactor selection and tuning	Utilizes specific metal ions (e.g., Mg^2+^, Zn^2+^, Mn^2+^) to stabilize structure and catalysis	Enhances catalytic rate and activity	Cofactor optimization may improve contrast between matched and mismatched substrates	Metal‐ion identity and concentration influence DNAzyme folding and catalytic activity	[89, 90, 91]
Reaction Conditions	Controls temperature and ionic strength to favor specific hybridization	Enhances cleavage kinetics at optimal temperature	May reduce nonspecific hybridization under optimized conditions	Optimize reaction temperature and ionic strength for the specific DNAzyme–substrate system	[89, 91, 92]
Assay Design	Integrates amplification (e.g., RCA) and fluorogenic substrates	Amplifies weak target signals to detectable levels	Target‐dependent cleavage produces fluorescence; nonspecific background must be controlled	RCA‐based amplification; fluorophore–quencher readouts (e.g., FAM/BHQ)	[28, 87]
Structural and thermodynamic optimization	Adjusts duplex stability (Δ*G*) and reduces interference from secondary structures	Adjusts duplex stability (Δ*G*) and reduces interference from secondary structures	Adjusts duplex stability (Δ*G*) and reduces interference from secondary structures	Adjusts duplex stability (Δ*G*) and reduces interference from secondary structures	[89, 90, 93]
Computational and in silico design	Predicts DNAzyme folding, toehold dynamics, and hybridization energetics	Accelerates design of high‐activity DNAzymes	Supports the design of sequences with improved predicted mismatch discrimination by simulating SNP mismatch effects	Toehold‐mediated displacement modeling; free‐energy prediction	[4, 94, 95]
Experimental Validation	Tests assay performance using matched and mismatched targets under defined conditions	Tests assay performance using matched and mismatched targets under defined conditions	Tests assay performance using matched and mismatched targets under defined conditions	Tests assay performance using matched and mismatched targets under defined conditions	[90, 93, 96]

## 9 Current Challenges

### 9.1 Stability

While DNA‐based sensors exhibit much greater stability than those made from RNA or proteins, they still do not match the durability of organic or polymer‐based sensors. DNAzyme sensors, which are typically under 100 nucleotides, are stable for several weeks at room temperature in aqueous solutions and can last for years when stored as dry powders. Even though the substrate includes a single ribonucleotide, it remains more stable than full RNA sequences, and DNAzymes in dry form can be preserved for over 9 months [17].

For in vitro applications, DNAzyme sensors in solution can often complete detection within 2–15 min, so stability is typically less problematic. However, in cellular and in vivo environments, where delivery and imaging may require much longer times, nuclease activity poses a major threat to stability. Attaching signaling molecules such as fluorophores or quenchers to the DNAzyme ends or incorporating modifications such as phosphorothioate bonds and inverted nucleotides can help protect against exonuclease degradation. Additionally, DNAzymes can adopt compact three‐dimensional structures that may reduce accessibility to both exo‐ and endonucleases compared with linear DNA strands [17]. In cases where stability is particularly important, DNAzymes can be selected or optimized for resistance to degradation in cellular extracts or human serum. Inactive DNAzyme controls should also be included to confirm that observed cleavage results from DNAzyme activity rather than nonspecific degradation [17].

### 9.2 Target Accessibility and Structural Constraints

Target accessibility is another challenge that researchers face when designing DNAzyme‐based sensors. For example, biological RNAs frequently form stable secondary and tertiary structures that can limit or prevent access to DNAzyme recognition or cleavage sites, thereby reducing hybridization efficiency and catalytic activity [12, 97]. Moreover, in double‐stranded DNA amplicons, the target sequence is sequestered within a duplex and may require strand separation, strand invasion, or other target‐exposure strategies before efficient DNAzyme recognition can occur. These structural constraints create a greater need for careful optimization of target‐site selection, recognition‐arm length and sequence, cofactor concentration, ionic strength, temperature, and reaction time.

### 9.3 Temperature

The catalytic beacon method’s sensitivity to temperature variations arises because it depends on changes in melting temperature due to metal‐dependent cleavage. This can impact the performance of DNAzyme sensors, particularly in applications like environmental monitoring and medical diagnostics, where ambient temperatures can vary widely. However, this issue is less problematic for cellular and in vivo sensing, given that physiological temperatures remain relatively stable [17]. To tackle this challenge, Nagraj and co‐workers introduced deliberate mismatches in the binding arms of the DNAzyme [92]. These mismatches do not compromise DNAzyme activity or metal selectivity but help adjust the sensor’s operating temperature range. Additionally, by combining two DNAzymes optimized for different temperature ranges‐one for high temperatures and one for low‐they created a DNAzyme‐based sensing system that maintains functionality across a temperature range of 4–30 °C [92].

### 9.4 Matrix Effects and Reproducibility

Performance in complex biological samples remains one of the least well‐characterized aspects of DNAzyme‐based SNP detection. Reproducibility across heterogeneous patient samples remains a central concern as endogenous nucleases, variable ion concentrations, and competing nucleic acid sequences in clinical specimens can substantially diminish DNAzyme catalytic efficiency and specificity. Standardization of assay conditions, particularly metal‐ion cofactor concentrations and reaction temperatures, to achieve robust performance across diverse sample types can be difficult. Reporting assay performance in the intended sample matrix, rather than in buffer alone, is therefore essential for meaningful comparison between platforms [12, 17, 28, 55].

## 10 Future Perspectives

The integration of DNAzyme‐based SNP detection technologies into clinical practice and research represents a paradigm shift in healthcare. These versatile and highly specific catalytic molecules address critical challenges in precision medicine, genomics, and preventive care. Their ability to discriminate single‐nucleotide changes with high selectivity provides clinicians and researchers with a programmable tool for understanding and managing genetic variability [55]. As technology matures, DNAzyme‐based assays have the potential to complement existing diagnostic technologies, enabling personalized therapeutic strategies, large‐scale genomic studies, and population‐wide screening initiatives. Their adaptability to various platforms, including point‐of‐care devices and automated workflows, further enhances their practicality and reach [55]. With ongoing advancements in nanotechnology and computational modeling, the future of DNAzyme‐based SNP detection promises even greater sensitivity, specificity, and scalability, and with additional research and development, these platforms could become a valuable asset in molecular medicine.

### 10.1 Personalized Medicine

In the field of personalized medicine, DNAzymes offer transformative potential by providing precise tools for SNP identification. These catalytic DNA molecules can be engineered to recognize and cleave sequences containing specific SNPs, making them powerful tools for creating personalized treatment plans [55]. For instance, DNAzyme‐based detection of pharmacogenetically relevant SNPs, such as those in cytochrome P450 enzymes, may contribute to predicting an individual’s response to medications [1, 96]. Such genotyping may help inform the selection of appropriate therapies and dosing strategies that are both effective and safe, avoiding adverse reactions or inefficacies associated with certain genetic profiles. Additionally, DNAzymes have shown promise in detecting SNPs linked to drug resistance in cancer therapy. Genetic variants and mutations in cancer‐associated genes, including EGFR, can influence diagnosis, prognosis, and therapeutic stratification. DNAzyme‐based platforms capable of discriminating clinically relevant sequence variants may therefore contribute to precision oncology [26, 83].

### 10.2 Genomics

DNAzyme‐based assays may provide useful tools for SNP analysis in genomics research, particularly for studying SNPs and their impact on genetic diversity and disease susceptibility. SNPs are the most common form of genetic variation, often used as markers for traits and diseases. By enabling the rapid, accurate, and cost‐effective detection of SNPs, DNAzyme‐based assays could support population‐scale SNP studies aimed at uncovering these genetic patterns [26, 28]. In principle, scalable SNP assays could facilitate genotyping across large and genetically diverse cohorts, revealing variations that contribute to differences in disease risk, drug response, or physical traits. These studies can help researchers investigate genotype–phenotype relationships and identify disease‐associated variants [26, 28, 98]. Furthermore, SNP detection can facilitate investigation of regulatory variants in noncoding genomic regions and their potential effects on gene regulation and disease susceptibility [99].

### 10.3 Disease Prevention

Accurate SNP detection may contribute to genetic risk assessment and preventive health strategies [26, 28]. Many genetic disorders and chronic diseases are influenced by SNPs, and identifying these variations before symptoms arise allows for timely intervention [26]. Because SNPs are associated with susceptibility to several cancers, accurate SNP detection may contribute to genetic risk assessment and screening strategies [38, 39, 40]. Identification of APOE and other AD‐associated variants can contribute to genetic risk assessment and may inform individualized risk assessment, prevention, and monitoring strategies [100].

Moreover, DNAzyme technology can be integrated into population health programs to identify at‐risk individuals early. For example, SNPs in genes like FTO, linked to obesity, or MTHFR, affecting folate metabolism, can guide nutritional and lifestyle recommendations tailored to an individual’s genetic profile [101].

By making genetic screening more accessible and accurate, DNAzymes empower healthcare systems to shift from reactive treatment to proactive prevention, reducing the burden of genetic diseases and improving long‐term health outcomes for individuals and populations.

### 10.4 Clinical Translation

The ddREVEALR platform demonstrated 95% positive predictive agreement in an evaluation of 20 nasopharyngeal swab samples for amplification‐free SARS‐CoV‐2 detection [59]. Despite encouraging proof‐of‐concept studies, DNAzyme‐based SNP assays have not yet achieved widespread clinical or regulatory adoption. Several barriers account for this gap. Beyond the matrix effects and standardization challenges discussed above, manufacturing scalability and shelf stability also present regulatory hurdles that have not yet been systematically addressed in the literature. Bridging this gap will require prospective validation studies in clinically relevant cohorts, harmonization with existing diagnostic workflows such as next‐generation sequencing or digital PCR, and direct engagement with regulatory science frameworks. These steps, though demanding, are achievable and represent the logical next frontier for the field.

## 11 Summary and Outlook

DNAzymes may offer a transformative approach to SNP detection by addressing key limitations of traditional methods like PCR and RFLP. Their high specificity for single‐nucleotide variations, coupled with their adaptability for various detection platforms such as fluorescence, colorimetric, and electrochemical systems, makes them a promising tool for molecular diagnostics. DNAzyme‐based sensors could provide rapid, cost‐effective, and highly sensitive solutions, positioning them as candidates that, with additional research and development, could become valuable assets in clinical applications. As detailed in this review, recent advancements in optimizing DNAzyme design, including binding arm engineering, cofactor selection, and computational modeling, have further enhanced their accuracy and efficiency. Signal amplification strategies, such as RCA and HCR, have substantially enhanced signal generation and analytical sensitivity in DNAzyme‐based assays.

The integration of DNAzyme technology into genomics, personalized medicine, and disease prevention initiatives offers vast potential. These tools could support genetic risk assessment, therapeutic stratification, and large‐scale genetic studies. As research continues to refine their stability, functionality, and application breadth, DNAzymes have the potential to contribute to advances in precision healthcare and improve outcomes for patients and populations alike. However, despite significant progress in the field, practical translation remains limited by mismatch discrimination, nuclease stability, matrix effects, assay reproducibility, and the lack of standardized benchmarking across platforms. Future progress will depend on integrating structure‐guided and computational design with robust controls, multiplexed detection, simplified sample preparation, and validation in clinically relevant samples. Improved understanding of catalytic mechanisms and more systematic comparison with established SNP‐‍detection methods will be essential for moving DNAzyme‐based sensors from proof‐of‐concept demonstrations toward reliable point‐of‐care and personalized diagnostic applications.

## Funding

KD is funded by the National Institute of General Medical Sciences of the National Institutes of Health under award 1 R16GM149496, RCMI award 1U54MD020891, the National Science Foundation under award 2302452, and by the U.S. Army Combat Capabilities Development Command – Soldier Center (cooperative agreement W911QY2220005).

## Conflicts of Interest

K.D. is a cofounder of Exoverse Solutions, LLC. The company had no role in the design or writing of this paper and has no financial interest in its outcome.

## Data Availability

Data sharing is not applicable to this article as no datasets were generated or analyzed during the current study.
